# Differential expression of antiviral and immune-related genes in individuals with COVID-19 asymptomatic or with mild symptoms

**DOI:** 10.3389/fcimb.2023.1173213

**Published:** 2023-06-14

**Authors:** Malena Gajate-Arenas, Omar García-Pérez, Javier Chao-Pellicer, Angélica Domínguez-De-Barros, Roberto Dorta-Guerra, Jacob Lorenzo-Morales, Elizabeth Córdoba-Lanus

**Affiliations:** ^1^ Instituto Universitario de Enfermedades Tropicales y Salud Pública de Canarias (IUETSPC), Universidad de La Laguna, La Laguna, Spain; ^2^ Centro de Investigación Biomédica en Red de Enfermedades Infecciosas (CIBERINFEC), Instituto de Salud Carlos III, Madrid, Spain; ^3^ Departamento de Matemáticas, Estadística e Investigación Operativa, Facultad de Ciencias, Sección de Matemáticas, Universidad de La Laguna, La Laguna, Spain; ^4^ Departamento de Obstetricia y Ginecología, Pediatría, Medicina Preventiva y Salud Pública, Toxicología, Medicina Legal y Forense y Parasitología, Facultad de Ciencias de la Salud, Universidad de La Laguna, La Laguna, Spain

**Keywords:** COVID-19, SARS-CoV-2, upper airway, gene expression, asymptomatic, antiviral genes, immune response

## Abstract

COVID-19 is characterized by a wide range of symptoms where the genetic background plays a key role in SARS-CoV-2 infection. In this study, the relative expression of *IRF9, CCL5, IFI6, TGFB1, IL1B, OAS1*, and *TFRC* genes (related to immunity and antiviral activity) was analyzed in upper airway samples from 127 individuals (97 COVID-19 positive and 30 controls) by using a two-step RT-PCR. All genes excepting *IL1B* (p=0.878) showed a significantly higher expression (p<0.005) in COVID-19 cases than in the samples from the control group suggesting that in asymptomatic-mild cases antiviral and immune system cells recruitment gene expression is being promoted. Moreover, *IFI6* (p=0.002) and *OAS1* (p=0.044) were upregulated in cases with high viral loads, which could be related to protection against severe forms of this viral infection. In addition, a higher frequency (68.7%) of individuals infected with the Omicron variant presented higher viral load values of infection when compared to individuals infected with other variants (p<0.001). Furthermore, an increased expression of *IRF9* (p<0.001)*, IFI6* (p<0.001), *OAS1* (p=0.011)*, CCL5*, (p=0.003) and *TGFB1* (p<0.001) genes was observed in individuals infected with SARS-CoV-2 wildtype virus, which might be due to immune response evasion of the viral variants and/or vaccination. The obtained results indicate a protective role of *IFI6*, *OAS1* and *IRF9* in asymptomatic -mild cases of SARS-CoV-2 infection while the role of *TGFB1* and *CCL5* in the pathogenesis of the disease is still unclear. The importance of studying the dysregulation of immune genes in relation to the infective variant is stand out in this study.

## Introduction

Coronavirus disease 2019 (COVID-19), caused by the severe acute respiratory syndrome coronavirus 2 (SARS-CoV-2), emerged in Wuhan (China) in December 2019. Its efficient transmission and the wide human mobility worldwide promoted the fast spread of COVID-19, with the World Health Organization (WHO) declaring it a pandemic on 11 March 2020. Since then, COVID-19 has become a world public health issue causing dramatic health and economic issues in many countries (Wiersinga et al., 2020; Deng et al., 2021; Hu et al., 2021).

Coronaviruses constitute a family of large, enveloped, single-stranded RNA viruses, well-known for causing respiratory infections. Two coronaviruses caused epidemic outbreaks, the severe acute respiratory syndrome (SARS) in 2002-2003 (China) and the Middle East respiratory syndrome (MERS) in 2012 (Arabian Peninsula). The origin of SARS-CoV-2 remains unclear although a zoonotic event is suspected focusing on bats as natural reservoirs of this virus (Ovsyannikova et al., 2020; Wiersinga et al., 2020; V’kovski et al., 2021).

SARS-CoV-2 is mainly transmitted by droplets expelled during talking, coughing, or sneezing (Wiersinga et al., 2020). When humans are infected by SARS-CoV-2, the virus main target are the airway cells. Moreover, the virus is able to invades cells after the binding of the S protein to the ACE2 (angiotensin-converting enzyme 2) receptor, while TMPRSS2 (transmembrane serine protease 2) promotes viral uptake by cleaving ACE2. After that, SARS-CoV-2 starts to replicate in epithelial cells from the respiratory tract and then migrates to the lungs. In this scenario, strong immune responses are promoted which can trigger off damage in the lung instead of relieving the infection (Wiersinga et al., 2020; Deng et al., 2021; Hu et al., 2021; Kumar et al., 2021; Yang and Rao, 2021). The cytokine storm is the main characteristic of this immune response, resulting from an acute increase of proinflammatory cytokines which promotes the influx of immune cells like T cells or macrophages from circulation to the lungs. As a result of the acute lung damage, patients may progress to develop respiratory distress syndrome and respiratory failure followed by multi-organ failure. Cytokine storm is considered the main cause of mortality in COVID-19 cases (Ragab et al., 2020).

People with COVID-19 have a wide range of symptoms, from asymptomatic or mild symptoms (the most common symptoms are fatigue, dry cough, and fever) to severe cases that need hospitalization or intensive care. Most of the severe symptoms affect people over 60 years, especially men. Biological and genetic factors may be involved in these facts (Wiersinga et al., 2020; Hu et al., 2021; Inde et al., 2021; Tharakan et al., 2022). Clinical comorbidities like diabetes, obesity, cardiovascular pathologies, etc. can increase the risk of developing an acute respiratory distress syndrome too (Casanova and Su, 2020; Debnath et al., 2020). However, the age and clinical features are not enough to explain the phenotypic variability of COVID-19. The genetic variability of each individual is playing a crucial role in the susceptibility and pathogenesis of the virus (Amati et al., 2020). The gene expression analysis has been useful for detecting deficiencies in other respiratory viruses related to worse disease outcomes. There is evidence to show the same with COVID-19, although studies were mainly performed on severe cases of the disease, which does not contribute to explain the whole spectrum of heterogeneity in the disease (Amati et al., 2020; Casanova and Su, 2020; Mick et al., 2020).

The study of gene expression on pathways related to the pathogenesis of SARS-CoV-2 and the immune system response could shed some light on why certain people develop severe forms of the disease and why others do not. In upper airways takes place infection, replication, and a local immune response, being these the first target of SARS-CoV-2 before reaching the lungs (Huang et al., 2021). Moreover, the analyses of different tissue samples show a differential response to SARS-CoV-2 infection among tissues, likely because of a local immune response but relating to the disease severity at systemic level (Gómez-Carballa et al., 2022).The main objective of the study was to determine any alteration in inflammatory or immune Genes expression in upper airways samples from individuals with COVID-19 asymptomatic or with mild symptoms in contrast to controls that were not infected with SARS-CoV-2. The identification of potential genetic biomarkers related to COVID-19 development may improve the patient diagnosis and prognosis, and will increase the disease knowledge (Ovsyannikova et al., 2020).

## Materials and methods

### Individuals included in the study

Upper airway samples that included nasopharyngeal and oropharyngeal/saliva were collected from 97 cases with a molecular diagnosis of COVID-19, which were recruited from a population screening study at University of La Laguna and private laboratories in Tenerife, Spain. The samples analyzed were collected during the first seven days of infection or at the beginning of symptoms. The study included male and female individuals of a wide range of ages and focused on asymptomatic and mild symptomatic infected individuals. Subjects were full vaccinated (with 2 or 3 doses) at the time of sample collection, except for individuals infected with the wildtype variant, since COVID-19 vaccines were not available at that period. Thirty individuals from the general population with a negative diagnosis of COVID-19 were also included as a control group. These subjects were analyzed for SARS-CoV-2 infection by qPCR at least in two different time points within five days. A negative report of having suffered any viral infection during the last 2 months before sample collection was required for participate. Subjects with underlying diseases or those undergoing medical treatment were excluded from the study. The study was approved by the ethical committee board and written informed consent was obtained from all participants (CHUC B1947). This study was conducted in accordance with the Declaration of Helsinki.

### Selection of genes

Genes were selected based on existing literature where the type of sample, methodology of the study, and relevant results were considered. The search was focused on genes related to the host antiviral and immune response that have been reported to be differentially expressed in different type of samples, particularly upper airways, in COVID-19 cases of different severity. *TFRC* was initially chosen as a candidate reference gene, although its expression has found to be altered in the samples of study and subsequently been analyzed as another target gene.

### Gene expression study

The RNA extraction was carried out using Maxwell^R^ 16 Viral Total Nucleic Acid Purification Kit (Promega) and Mag-Bind^®^ Viral RNA XPress Kit (Omega Bio-Tek), and quality was evaluated by NanoDrop Lite (ThermoScientific, USA).

The relative gene expression analysis was set up in a two-step RT-PCR. First, RNA was retrotranscribed into cDNA using the High-Capacity cDNA Reverse Transcription Kit (Applied Biosystems, USA) following the manufacturer’s instructions. In a second step, a qPCR was performed by using the: TaqMan™ Gene Expression Master Mix and TaqMan™ Gene Expression Assays (ThermoFisher Scientific, Applied Biosystem, USA) (**Supplementary Table 1**), and 2.5 µl of cDNA in a final volume reaction of 10 ul. The reaction was performed in a real-time qPCR machine QuantStudio 5 (ThermoFisher Scientific, Applied Biosystem, USA). Each reaction was performed in duplicate, setting up the experiment in 40 cycles. For data normalization, the *ACTB* housekeeping gene was used. The relative expression analysis of the target genes was determined using the comparative threshold method 2−ΔΔCt (Kumar et al., 2022).

### Statistical analysis

The viral load analysis was ranged based on the Ct values that resulted from the qPCR diagnostic test: a low load (Ct>30), medium load (24>Ct>30), and high load (Ct<24). The expression data distribution was analyzed by the Kolmogorov-Smirnov test. Data were normalized using the method of the log two-fold and absolute gene-wise changes in expression. Non-parametric tests, the Mann-Whitney U test, and the Kruskal-Wallis test were used for group comparisons. The correlation between variables was analyzed by Spearman’s rank correlation coefficient. Chi-squared test was performed to determine differences in gene expression frequencies between groups. Principal component analysis (PCA) was performed for dimension reduction and better interpretability of the data. For applying PCA, the data must achieve some requirements: multiple variables must be significatively related, and the determinant of the correlation matrix must be close to zero. The sampling adequacy is shown by the Kaiser-Meyer-Olkin Measure and for knowing data suitability for reduction Bartlett’s test of sphericity was performed. For all analyses, significance was set at p<0.05. The statistical analysis was performed using SPSS v. 25 (IBM Corp, USA) and GraphPad Prims v. 9.4.1 (Dotmatics, UK) software.

## Results

A total of 127 individuals were analyzed in the present study. From these, 97 individuals were positive when tested for COVID-19 and 30 individuals with a negative record were included as controls. The main characteristics of these subjects are shown in **Table 1**. Infected individuals presented mild symptoms (fever, cough, headache, etc.) (33.37%) or were asymptomatic (66.67%). The COVID-19-positive individuals were 50.52% of men with a mean age of 42 years and were age-matched with controls without the infection.

**Table 1:** Demographic and clinical characteristics of the individuals with COVID-19 included in the study.

Variables	COVID-19 cases n=97
**Age (mean ± SD)**	41.93 ± 16.94
Sex (%)	
**• Women**	49.48
**• Men**	50.52
Type of sample (%)	
**• Nasopharyngeal**	77.32
**• Oropharyngeal/saliva**	22.68
SARS-CoV-2 variant ^‡^(%)	
**• Wild type**	42.27
**• B.1.1.7**	4.12
**• B.1.617.2**	11.34
**• B.1.1.529**	42.27

**^‡^** B.1.17: Alpha variant; B.1.617.2: Delta variant; B.1.1.529: Omicron variant. https://www.who.int/activities/tracking-SARS-CoV-2-variants. Pango nomenclature was used (Rambaut et al., 2020).

Within the infected individuals, 34% presented a high viral load, 34% medium load, and 32% a low load (average viral load was Ct= 25.67 ± 6.56). Four SARS-CoV-2 variants were detected as responsible for infection within the recruited individuals as follows: forty-one were infected with the original virus from Wuhan (index virus), four with variant B.1.1.7, eleven with B.1.617.2, and forty-one corresponded to individuals infected with B.1.1.529 (**Table 1**).

**Figure 1:**
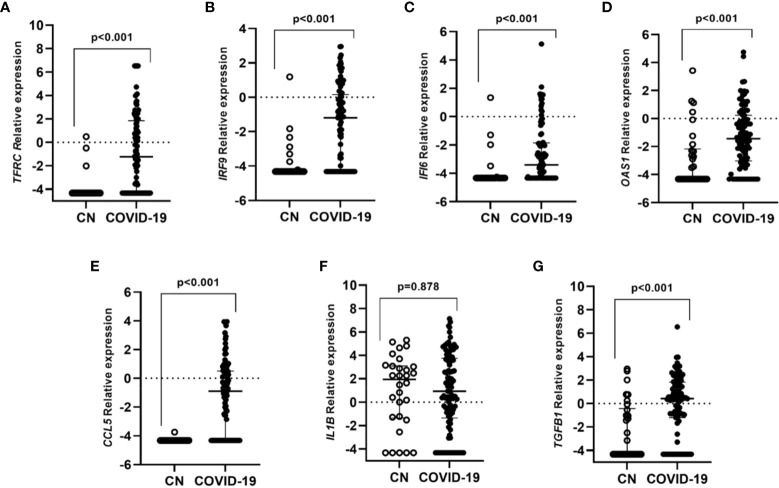
Differential gene expression in individuals with COVID-19 in contrast to controls subjects without infection. Lines represent the median with an interquartile range. **(A)** TFRC, **(B)** IRF9, **(C)** IFI6, **(D)** OAS1, **(E)** CCL5, **(F)** IL1B and **(G)** TGFB1. p-values<0.05 were considered significant.

The expression of *TFRC, IRF9, IFI6, OAS1, CCL5, IL1B*, and *TGFB1* genes was determined in all the participants. All genes were highly expressed in COVID-19 individuals when compared to the control group except for *IL1B* (**Figure 1**).

**Figure 2:**
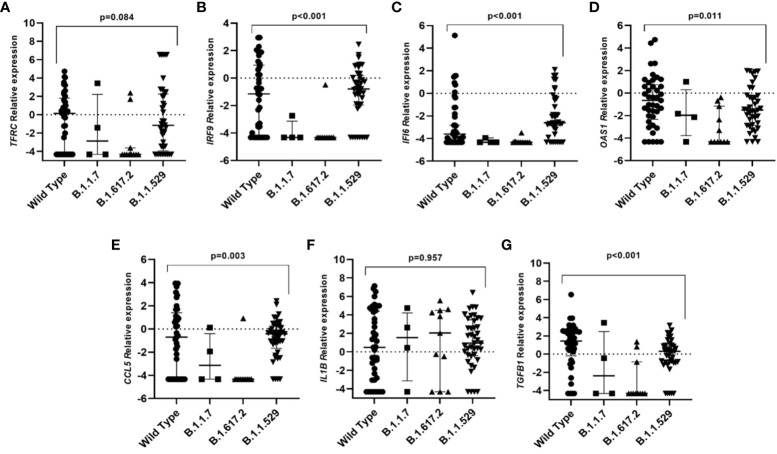
Differential gene expression among individuals infected with different SARS-CoV-2 variants. Lines represent the median with an interquartile range. **(A)** TFRC, **(B)** IRF9, **(C)** IFI6, **(D)** OAS1, **(E)** CCL5, **(F)** IL1B and **(G)** TGFB1. p-values<0.05 were considered significant.

When differences in gene expression among SARS-CoV-2 variants were explored we observed that individuals infected with the original variant presented higher expression levels of *IRF9, IFI6, OAS1, CCL5*, and *TGFB1* genes than individuals infected with other variants (**Figure 2**).

### Gene expression and viral load

**Figure 3:**
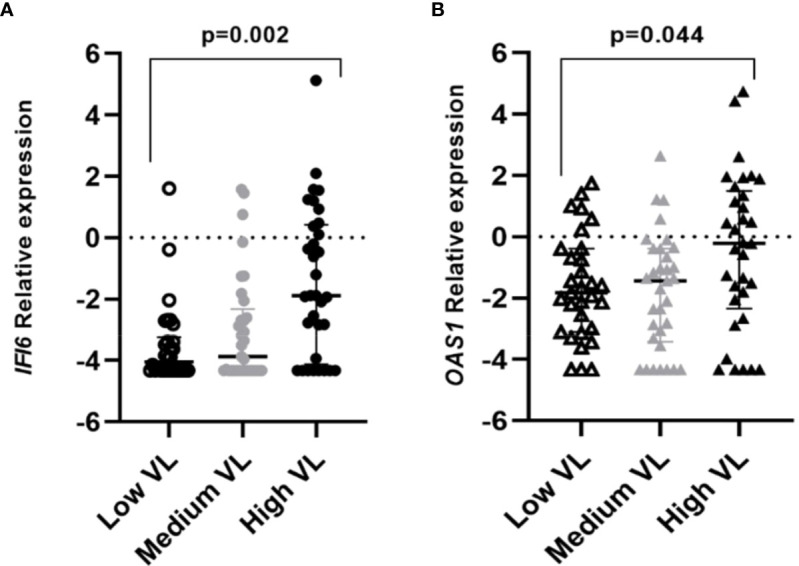
Differential gene expression among individuals with different viral loads (VL=Viral Load). Lines represent the median with an interquartile range. **(A)** IFI6, **(B)** OAS1. p-values<0.05 were considered significant.

A higher frequency of individuals infected with the B.1.1.529 variant presented higher viral load values of infection (Ct<24) when compared to individuals infected with the original variant (68.7% vs 32.3% respectively; p<0.001). Significative differences were found between the expression of the studied genes and the viral load within COVID-19 cases. Individuals that presented a high viral load also had increased expression levels of *OAS1* (p=0.011) and *IFI6* (p<0.001) genes (**Figure 3**, **Supplementary Figure 1**).

In relation to sex, infected men were found to highly express *OAS1* when compared to women (p=0.026), but among healthy individuals non-significative result was found. No significant relationship was found between sex and other variables, such as viral load (p=0.753) or the SARS-CoV-2 variants (p=0.387).

### Analysis of gene expression through principal component

For the principal component analysis, criteria for valid results were first tested as adequate. Multiple variables were significatively related to each other (**Supplementary Table 2**) and the correlation matrix was determined as 0.043. The sampling adequacy was detected by the Kaiser-Meyer-Olkin Measure (0.834) and Bartlett’s test of sphericity (p<0.001).

Two components based on eigenvalues > 1 were selected. The two resulting components explained 68.92% of the total variance and showed that most of the genes were related to component 1, with *IL1B* related to component 2 (**Supplementary Figure 2**).

## Discussion

SARS-CoV-2 can cause a wide range of symptoms, and elder and previous pathologies often are indicators of worse outcomes, however, other factors may play an important role (Jajou et al., 2022).

In the present study, the differential expression of a profile of immune-related and antiviral genes was evaluated in upper airway samples in relation to asymptomatic and mild COVID-19 cases.

All the studied genes were differently expressed between SARS-CoV-2 individuals and the healthy control group, except for *IL1B*, which was confirmed by the PCA analysis. The first component grouped genes that were overexpressed in COVID-19 cases when compared to healthy controls. These genes, related to the immune system, *IFI6, IRF9*, and *OAS1* are interferon-stimulated genes (ISG) that play an important role in pathogen control (Malterer et al., 2014; Meyer et al., 2015).

IFI6 is a mitochondria-target protein that participates in apoptosis regulation and its expression has been related to viral inhibition (Meyer et al., 2015; Kuroda et al., 2020). The higher expression of *IFI6* observed in infected individuals is in agreement with another study which finds its upregulation in SARS-CoV-2 infections when compared with other respiratory viruses (Mick et al., 2020). The expression of *IFI6* does not inhibit viral replication by itself but might enhance the antiviral effect of IFN-α (Meyer et al., 2015).

*IRF9* expression has been related to multiple respiratory diseases and its deficiency is associated with worse outcomes of respiratory viral infections, including SARS-CoV-2 (Zhang et al., 2021). Our results showed a higher expression in COVID-19 cases, that included only asymptomatic or mild forms of the disease, then, the higher expression of *IRF9* may be related to better outcomes which is in accordance to other studies which found *IRF9* deficient levels in severe cases (Zhang et al., 2021; Ziegler et al., 2021).

In the same way, *OAS1* was highly expressed in infected subjects. A higher expression of *OAS1* may be characteristic of mild-moderate COVID-19 patients while the expression deficiency has been related to a higher risk of severe COVID-19 in other studies (Ziegler et al., 2021; Banday et al., 2022) suggesting a protective effect in SARS-CoV-2 infection for *OAS1*. This gene was the only one dysregulated between sexes with higher levels of expression among men. *OAS1* presents different polymorphisms that can alter its expression levels, however, their analysis was beyond the scope of the present study (Zhou et al., 2021; Banday et al., 2022).

CCL5 is a chemoattractant for several immune cells like monocytes and natural killers. Our results are in agreement with Perez-Garcia et al. (2022) who reported that high levels of *CCL5* were associated with better outcomes in COVID-19 patients. However, other studies found a higher expression of *CCL5* in critical COVID-19 patients, suggesting a role prompting inflammation disorders (Montalvo Villalba et al., 2020; Ye et al., 2020). Studies on severe COVID-19 cases are needed to confirm this fact.

Our study found higher expression of *TGFB1* in the SARS-CoV-2 infected group in contrast to controls. *TGFB1* is the predominant isoform expressed in the immune system and plays a role in cell proliferation, differentiation, migration, and survival. It exists evidence of viruses that promote the expression of *TGFB1*, such as the neuraminidase of influenza A and influenza B (Li et al., 2006). Another example is the papain-like protease of SARS-CoV that induces the upregulation of *TGFB1* (Li et al., 2016). Furthermore, higher levels of *TGFB1* were reported to be associated with pulmonary fibrosis, including COVID-19 patients (Li et al., 2016; Florindo et al., 2020). Studies that include mild to severe and critical cases are needed to confirm if the upregulation of *TGFB1* enhances poor COVID-19 outcomes as may be pulmonary fibrosis development.

*IL1B* presented similar levels of expression between cases and controls. Mick et al. (2020) suggested that SARS-CoV-2 infection promotes a low expression of *IL1B* compared to other respiratory viruses in the early course of the infection. Similarly, a recent study did not detect any significative differential *IL1B* expression in severe cases (Gómez-Carballa et al., 2022). *IL1B* is produced by the inflammasome complex which is particularly representative in the macrophage population, and though inflammasome pathways might be non-responsive during SARS-CoV-2 infection (Mick et al., 2020; Gómez-Carballa et al., 2022). However, IL1B is a pro-inflammatory cytokine that contributes to developing ARDS (Acute Respiratory Distress Syndrome) in SARS and MERS infections, and higher expression levels of *IL1B* have been found in severe cases. Macrophage populations could overexpress pro-inflammatory mediators which promote excessive inflammation (Chua et al., 2020; Ye et al., 2020) in severe forms of the disease, not in mild forms or asymptomatic cases as confirmed in the present study.

*TFRC* was analyzed as a candidate reference gene, however, we found a higher expression of this gene between SARS-CoV-2 infected individuals and the healthy control group. *TFRC* might have an indirect relationship with *ACE2* which could explain its overexpression in COVID-19 cases (Wicik et al., 2020).

The viral load has become one of the most appealing points in research, due to its relationship with the transmission of the virus. Moreover, a higher viral load in the upper respiratory tract has been associated to comorbidities, aging, and severe forms of the disease (Maltezou et al., 2021; Jajou et al., 2022). In the present study, it was found that *OAS1* and *IFI6* were overexpressed in individuals with a high viral load of infection. Both genes are inducible by interferon; a previous study found out higher levels of interferon-responsive genes in individuals with high viral loads (Lieberman et al., 2020). *OAS1* encodes for an antiviral enzyme that negatively affects viral replication. OAS proteins can identify viral RNA, synthesize 2’-5’ oligoadenylates, and then bind and activate RNase L degrading the viral RNA (Kristiansen et al., 2011; Padariya et al., 2021). High viral load involves a greater amount of viral particles, which entails an increase of *OAS1* activity, and translates into higher expression levels that may contribute to the protection against the severe forms of the disease (Wickenhagen et al., 2021). In the same way, the *IFI6* gene takes part in the antiviral response (Schoggins et al., 2011), so its overexpression in individuals with high viral load could be related to mechanisms that reduce the viral replication (Meyer et al., 2015; Dukhovny et al., 2019; Kuroda et al., 2020).

Different variants have spread during the pandemic and each variant has mutations that provide adaptations that allow them to take advantage over other lineages (Chen et al., 2022). B.1.1.529 variant (Omicron) showed higher ratios of viral load than the other variants which prompt its higher transmissibility as has been demonstrated in other studies (He et al., 2021). An increased expression of genes related to the immune system in relation to the SARS-CoV-2 wild-type infection was observed in the present study and might have two reasons. First, several studies have shown that the new variants that surged after the original one can evade the immune response and escape from the immune system (Garcia-Beltran et al., 2021; Zhang L. et al., 2022), showing in consequence the downregulation of these genes in individuals infected with the mutant variants. Second, individuals who were infected with the wild-type virus were not yet vaccinated, so they developed a different immune response facing individuals who were later vaccinated and subsequently infected with a mutant SARS-CoV-2 variant (Cohen and Burbelo, 2021; Harvey et al., 2021; Netea and Li, 2021; Planas et al., 2022; Zhang Z. et al., 2022).

The present study has some limitations. First, our study focused on asymptomatic and mild COVID-19 cases, excluding severe cases which might be necessary to confirm some of the present findings. Secondly, wild-type (index virus) and Omicron SARS-CoV-2 variants were the predominant ones in this study. Few individuals infected with B.1.1.7 and B.1.617.2 were included.

In conclusion, SARS-CoV-2 infection increased the expression of genes related to the immune system in asymptomatic and mild symptomatic individuals. While a protective effect of *OAS1*, *IRF9*, and *IFI6* in asymptomatic and mild COVID-19 is confirmed, the role of *TGFB1* and *CCL5* is still controversial. *OAS1* and *IFI6* were overexpressed in individuals with a high viral load of infection. Importantly, the appearance of new variants might change our vision of SARS-CoV-2 pathogenesis hence further studies in this field are necessary.

## Data availability statement

The original contributions presented in the study are included in the article/**Supplementary Material**, further inquiries can be directed to the corresponding author/s.

## Ethics statement

The studies involving human participants were reviewed and approved by Ethical committee board Hospital Universitario de Canarias (CHUC B1947). The patients/participants provided their written informed consent to participate in this study.

## Author contributions

EC-L and JL-M participated in the conception and design. MG-A, OG-P, JC-P, AD-D-B, JL-M and EC-L participated in experimentation and data acquisition. EC-L, JL-M and MG-A participated in the analysis and interpretation and drafting of the manuscript for important intellectual content and take responsibility for the integrity of the data and the accuracy of the data analysis. RD-G participated in data analysis and interpretation. All authors read and approved the final manuscript.

## References

[B1] Amati F. Vancheri C. Latini A. Colona V. L. Grelli S. D'Apice M. R.. (2020). ‘Expression profiles of the SARS-CoV-2 host invasion genes in nasopharyngeal and oropharyngeal swabs of COVID-19 patients’. Heliyon 6 (10), e05143. doi: 10.1016/j.heliyon.2020.e05143 33024851 PMC7528978

[B2] Banday A. R. Stanifer M. L. Florez-Vargas O. Onabajo O. O. Papenberg B. W. Zahoor M. A.. (2022). Genetic regulation of OAS1 nonsense-mediated decay underlies association with COVID-19 hospitalization in patients of European and African ancestries. Nat. Genet. 54, 1103–1116. doi: 10.1038/s41588-022-01113-z 35835913 PMC9355882

[B3] Casanova J.-L. Su H. C. (2020). A global effort to define the human genetics of protective immunity to SARS-CoV-2 infection. Cell 181 (6), 1194–1199. doi: 10.1016/j.cell.2020.05.016 32405102 PMC7218368

[B4] Chen K. W. K. Tsung-Ning Huang D. Huang L. M. (2022). SARS-CoV-2 variants – evolution, spike protein, and vaccines. Biomed. J. Chang Gung Univ. 45 (4), 573–579. doi: 10.1016/j.bj.2022.04.006 PMC9072773 35526825

[B5] Chua R. L. Lukassen S. Trump S. Hennig B. P. Wendisch D. Pott F.. (2020). COVID-19 severity correlates with airway epithelium–immune cell interactions identified by single-cell analysis. Nat. Biotechnol. Springer US 38 (8), 970–979. doi: 10.1038/s41587-020-0602-4 32591762

[B6] Cohen J. I. Burbelo P. D. (2021). Reinfection with SARS-CoV-2: implications for vaccines. Clin. Infect. Dis. 73 (11), E4223–E4228. doi: 10.1093/cid/ciaa1866 33338197 PMC7799323

[B7] Debnath M. Banerjee M. Berk M. (2020). Genetic gateways to COVID-19 infection: implications for risk, severity, and outcomes. FASEB J. 34 (7), 8787–8795. doi: 10.1096/fj.202001115R 32525600 PMC7300732

[B8] Deng H. Yan X. Yuan L. (2021). Human genetic basis of coronavirus disease 2019. Sig. Transduct. Target. Ther. 6 (1), 344. doi: 10.1038/s41392-021-00736-8 PMC8450706 34545062

[B9] Dukhovny A. Lamkiewicz K. Chen Q. Fricke M. Jabrane-Ferrat N. Marz M.. (2019). A CRISPR activation screen identifies genes that protect against zika virus infection. J. Virol. 93 (16), e00211–e00219. doi: 10.1128/jvi.00211-19 31142663 PMC6675891

[B10] Florindo H. F. Kleiner R. Vaskovich-Koubi D. Acúrcio R. C. Carreira B. Yeini E.. (2020). Immune-mediated approaches against COVID-19. Nat. Nanotechnol. Springer US 15 (8), 630–645. doi: 10.1038/s41565-020-0732-3 PMC7355525 32661375

[B11] Garcia-Beltran W. F. Lam E. C. St Denis K. Nitido A. D. Garcia Z. H. Hauser B. M.. (2021). Multiple SARS-CoV-2 variants escape neutralization by vaccine-induced humoral immunity. Cell. Elsevier Inc 184 (9), 2372–2383.e9. doi: 10.1016/j.cell.2021.03.013 PMC7953441 33743213

[B12] Gómez-Carballa A. Rivero-Calle I. Pardo-Seco J. Gómez-Rial J. Rivero-Velasco C. Rodríguez-Núñez N.. (2022). A multi-tissue study of immune gene expression profiling highlights the key role of the nasal epithelium in COVID-19 severity. Environ. Res. 210, 112890. doi: 10.1016/j.envres.2022.112890 35202626 PMC8861187

[B13] Harvey R. A. Rassen J. A. Kabelac C. A. Turenne W. Leonard S. Klesh R.. (2021). Association of SARS-CoV-2 seropositive antibody test with risk of future infection. JAMA Internal Med. 181 (5), 672–679. doi: 10.1001/jamainternmed.2021.0366 33625463 PMC7905701

[B14] He X. Hong W. Pan X. Lu G. Wei X. (2021). SARS-CoV-2 omicron variant: characteristics and prevention. MedComm 2 (4), 838–845. doi: 10.1002/mco2.110 34957469 PMC8693031

[B15] Hu B. Guo H. Zhou P. Shi Z. L. (2021). Characteristics of SARS-CoV-2 and COVID-19. Nat. Rev. Microbiol. Springer US 19 (3), 141–154. doi: 10.1038/s41579-020-00459-7 PMC7537588 33024307

[B16] Huang N. Pérez P. Kato T. Mikami Y. Okuda K. Gilmore R. C.. (2021). SARS-CoV-2 infection of the oral cavity and saliva. Nat. Med. 27 (5), 892–903. doi: 10.1038/s41591-021-01296-8 33767405 PMC8240394

[B17] Inde Z. Croker B. A. Yapp C. Joshi G. N. Spetz J. Fraser C.. (2021). Age-dependent regulation of SARS-CoV-2 cell entry genes and cell death programs correlates with COVID-19 severity. Sci. Adv. 7 (34), 1–18. doi: 10.1126/sciadv.abf8609 PMC8373124 34407940

[B18] Jajou R. Mutsaers-van Oudheusden A. Verweij J. J. Rietveld A. Murk J. L. (2022). SARS-CoV-2 transmitters have more than three times higher viral loads than non-transmitters – practical use of viral load for disease control. J. Clin. Virol. Elsevier B.V. 150–151, 105131. doi: 10.1016/j.jcv.2022.105131 PMC8920080 35395500

[B19] Kristiansen H. Gad H. H. Eskildsen-Larsen S. Despres P. Hartmann R. (2011). The oligoadenylate synthetase family: an ancient protein family with multiple antiviral activities. J. Interferon Cytokine Res. 31 (1), 41–47. doi: 10.1089/jir.2010.0107 21142819

[B20] Kumar A. Prasoon P. Kumari C. Pareek V. Faiq M. A. Narayan R. K.. (2021). SARS-CoV-2-specific virulence factors in COVID-19. J. Med. Virol. 93 (3), 1343–1350. doi: 10.1002/jmv.26615 33085084

[B21] Kumar S. Ahmad A. Kushwaha N. Shokeen N. Negi S. Gautam K.. (2022). Selection of ideal reference genes for gene expression analysis in COVID-19 and mucormycosis.’, microbiology spectrum. Am. Soc. Microbiol. 10 (6), e0165622. doi: 10.1128/spectrum.01656-22 PMC9769637 36377893

[B22] Kuroda M. Halfmann P. J. Hill-Batorski L. Ozawa M. Lopes T. J. S. Neumann G.. (2020). Identification of interferon-stimulated genes that attenuate Ebola virus infection. Nat. Commun. Springer US 11, 2953. doi: 10.1038/s41467-020-16768-7 PMC7289892 32528005

[B23] Li M. O. Wan Y. Y. Sanjabi S. Robertson A. K. Flavell R. A. (2006). Transforming growth factor- β regulation of immune responses. Annu. Rev. Immunol. 24, 99–146. doi: 10.1146/annurev.immunol.24.021605.090737 16551245

[B24] Li S. W. Wang C. Y. Jou Y. J. Yang T. C. Huang S. H. Wan L.. (2016). SARS coronavirus papain-like protease induces egr-1-dependent up-regulation of TGF-β1 via ROS/p38 MAPK/STAT3 pathway. Sci. Rep. 6, 25754. doi: 10.1038/srep25754 27173006 PMC4865725

[B25] Lieberman N. A. P. Peddu V. Xie H. Shrestha L. Huang M. L. Mears M. C.. (2020). *In vivo* antiviral host transcriptional response to SARS-CoV-2 by viral load, sex, and age. PloS Biol. 18 (9), 1–17. doi: 10.1371/JOURNAL.PBIO.3000849 PMC7478592 32898168

[B26] Malterer M. B. Glass S. J. Newman J. P. (2014). Interferon-stimulated genes: a complex web of host defenses. Annu. Rev. Immunol. 32, 513–545. doi: 10.1038/jid.2014.371 24555472 PMC4313732

[B27] Maltezou H. C. Raftopoulos V. Vorou R. Papadima K. Mellou K. Spanakis N.. (2021). Association between upper respiratory tract viral load, comorbidities, disease severity, and outcome of patients with SARS-CoV-2 infection. J.?Infect. Dis. 223 (7), 1132–1138. doi: 10.1093/infdis/jiaa804 33388780 PMC7798974

[B28] Meyer K. Kwon Y. C. Liu S. Hagedorn C. H. Ray R. B. Ray R. (2015). Interferon-α inducible protein 6 impairs EGFR activation by CD81 and inhibits hepatitis c virus infection. Sci. Rep. 5, 9012. doi: 10.1038/srep09012 25757571 PMC4355636

[B29] Mick E. Kamm J. Pisco A. O. Ratnasiri K. Babik J. M. Castañeda G.. (2020). Upper airway gene expression reveals suppressed immune responses to SARS-CoV-2 compared with other respiratory viruses. Nat. Commun. 11 (1), 5854. doi: 10.1038/s41467-020-19587-y 33203890 PMC7673985

[B30] Montalvo Villalba M. C. Valdés Ramírez O. Muné Jiménez M. Arencibia Garcia A. Martinez Alfonso J. González Baéz G.. (2020). Interferon gamma, TGF-β1 and RANTES expression in upper airway samples from SARS-CoV-2 infected patients. Clin. Immunol. Elsevier 220 (August), 108576. doi: 10.1016/j.clim.2020.108576 PMC7455570 32866645

[B31] Netea M. G. Li Y. (2021). Immune memory in individuals with COVID-19. Nat. Cell Biol. 23 (6), 582–584. doi: 10.1038/s41556-021-00695-w 34108656

[B32] Ovsyannikova I. G. Haralambieva I. H. Crooke S. N. Poland G. A. Kennedy R. B. (2020). The role of host genetics in the immune response to SARS-CoV-2 and COVID-19 susceptibility and severity. Immunol. Rev. 296 (1), 205–219. doi: 10.1111/imr.12897 32658335 PMC7404857

[B33] Padariya M. Sznarkowska A. Kote S. Gómez-Herranz M. Mikac S. Pilch M.. (2021). Functional interfaces, biological pathways, and regulations of interferon-related dna damage resistance signature (Irds) genes. Biomolecules 11 (5), 622. doi: 10.3390/biom11050622 33922087 PMC8143464

[B34] Perez-Garcia F. Martin-Vicente M. Rojas-García R. L. Castilla-García L. Muñoz-Gomez M. J. Hervás Fernández I.. (2022). High SARS-CoV-2 viral load and low CCL5 expression levels in the upper respiratory tract are associated with COVID-19 severity. J. Infect. Dis. 225 (6), 977–982. doi: 10.1093/infdis/jiab604 34910814 PMC8754799

[B35] Planas D. Saunders N. Maes P. Guivel-Benhassine F. Planchais C. Buchrieser J.. (2022). Considerable escape of SARS-CoV-2 omicron to antibody neutralization. Nature. Springer US 602 (7898), 671–675. doi: 10.1038/s41586-021-04389-z 35016199

[B36] Ragab D. Salah Eldin H. Taeimah M. Khattab R. Salem R. (2020). The COVID-19 cytokine storm; what we know so far. Front. Immunol. 11. doi: 10.3389/fimmu.2020.01446 PMC7308649 32612617

[B37] Rambaut A. Holmes E. C. O'Toole Á. Hill V. McCrone J. T. Ruis C.. (2020). A dynamic nomenclature proposal for SARS-CoV-2 lineages to assist genomic epidemiology. Nat. Microbiol. Springer US 5 (11), 1403–1407. doi: 10.1038/s41564-020-0770-5 PMC7610519 32669681

[B38] Schoggins J. W. Wilson S. J. Panis M. Murphy M. Y. Jones C. T. Bieniasz P.. (2011). A diverse range of gene products are effectors of the type i interferon antiviral response. Nature. Nat. Publishing Group 472 (7344), 481–485. doi: 10.1038/nature09907 PMC3409588 21478870

[B39] Tharakan T. Khoo C. C. Giwercman A. Jayasena C. N. Sofikitis N. Salonia A.. (2022). Are sex disparities in COVID-19 a predictable outcome of failing men’s health provision? Nat. Rev. Urol. Springer US 19, 47–63. doi: 10.1038/s41585-021-00535-4 PMC8600906 34795426

[B40] V’kovski P. Kratzel A. Steiner S. Stalder H. Thiel V. (2021). Coronavirus biology and replication: implications for SARS-CoV-2. Nat. Rev. Microbiol. Springer US 19 (3), 155–170. doi: 10.1038/s41579-020-00468-6 PMC7592455 33116300

[B41] Wicik Z. Eyileten C. Jakubik D. Simões S. N. Martins D. C. Jr Pavão R.. (2020). ACE2 interaction networks in COVID-19: a physiological framework for prediction of outcome in patients with cardiovascular risk factors. J. Clin. Med. 9 (11), 3743. doi: 10.3390/jcm9113743 33233425 PMC7700637

[B42] Wickenhagen A. Sugrue E. Lytras S. Kuchi S. Noerenberg M. Turnbull M. L.. (2021). A prenylated dsRNA sensor protects against severe COVID-19. Science 374 (6567), eabj3624. doi: 10.1126/science.abj3624 34581622 PMC7612834

[B43] Wiersinga W. J. Rhodes A. Cheng A. C. Peacock S. J. Prescott H. C. (2020). Pathophysiology, transmission, diagnosis, and treatment of coronavirus disease 2019 (COVID-19) a review. J. Am. Med. Assoc. 324 (8), 782–793. doi: 10.1001/jama.2020.12839 32648899

[B44] Yang H. Rao Z. (2021). Structural biology of SARS-CoV-2 and implications for therapeutic development. Nat. Rev. Microbiol. Springer US 19 (11), 685–700. doi: 10.1038/s41579-021-00630-8 PMC8447893 34535791

[B45] Ye Q. Wang B. Mao J. (2020). The pathogenesis and treatment of the “Cytokine storm’’ in COVID-19. J. Infect. Elsevier Ltd 80 (6), 607–613. doi: 10.1016/j.jinf.2020.03.037 PMC7194613 32283152

[B46] Zhang Y. H. Li H. Zeng T. Chen L. Li Z. Huang T.. (2021). Identifying transcriptomic signatures and rules for SARS-CoV-2 infection. Front. Cell Dev. Biol. 8. doi: 10.3389/fcell.2020.627302 PMC7829664 33505977

[B47] Zhang L. Li Q. Liang Z. Li T. Liu S. Cui Q.. (2022). The significant immune escape of pseudotyped SARS-CoV-2 variant omicron. Emerg. Microbes Infect. 11 (1), 1–5. doi: 10.1080/22221751.2021.2017757 34890524 PMC8725892

[B48] Zhang Z. Mateus J. Coelho C. H. Dan J. M. Moderbacher C. R. Gálvez R. I.. (2022). Humoral and cellular immune memory to four COVID-19 vaccines. Cell 185 (14), 2434–2451. doi: 10.1016/j.cell.2022.05.022 35764089 PMC9135677

[B49] Zhou S. Butler-Laporte G. Nakanishi T. Morrison D. R. Afilalo J. Afilalo M.. (2021). A Neanderthal OAS1 isoform protects individuals of European ancestry against COVID-19 susceptibility and severity. Nat. Med. 27 (4), 659–667. doi: 10.1038/s41591-021-01281-1 33633408

[B50] Ziegler C. G. K. Miao V. N. Owings A. H. Navia A. W. Tang Y. Bromley J. D.. (2021). Impaired local intrinsic immunity to SARS-CoV-2 infection in severe COVID-19. Cell. Elsevier 184 (18), 4713–4733.e22. doi: 10.1016/j.cell.2021.07.023 PMC8299217 34352228

