# Supplementary material for: Differential expression of antiviral and immune-related genes in individuals with COVID-19 asymptomatic or with mild symptoms
Source: Front Cell Infect Microbiol. 2023 Jun 14;13:1173213. doi: 10.3389/fcimb.2023.1173213 (PMC10302728; doi:10.3389/fcimb.2023.1173213)

Supplementary Material

Supplementary Figures and Tables

Supplementary Table 1. Characteristics of the selected genes for the study.

Genes	Acronym	Reference sequence*	Assays reference**	Base pairs amplicon
**C-C Motif Chemokine Ligand 5**	*CCL5*	NM_001278736	Hs00982282_m1	70
**Interferon Alpha Inducible Protein 6**	*IFI6*	NM_002038	Hs00242571_m1	115
**Transforming Growth Factor Beta 1**	*TGFB1*	NM_000660	Hs00998133_m1	63
**Interleukin 1 Beta**	*IL1B*	NM_000576	Hs01555410_m1	91
**Interferon Regulatory Factor 9**	*IRF9*	NM_006084	Hs00196051_m1	66
**2'-5'-Oligoadenylate Synthetase 1**	*OAS1*	NM_001032409	Hs00973635_m1	82
**Transferrin Receptor**	*TFRC*	NM_001128148	Hs00951083_m1	63
**Actin Beta**	*ACTB*	NM_001101	Hs01060665_g1	63

*Reference sequence from the GenBank database, NCBI (National Center for Biotechnology Information), NIH (National Library of Medicine), USA.

** Assays used for gene expression detection (Thermofisher Scientific, USA).

Supplementary Table 2. Principal component analysis (PCA). Multiple variables relation.

	**1º PCA**			**2º PCA**	
**Genes**	**Component 1**	**Component 2**	**Communalities**	**Component 1**	**Communalities**

TFRC	0.773	-0.241	0.656	0.781	0.610
IRF9	0.891	-0.047	0.795	0.890	0.792
IFI6	0.777	-0.013	0.604	0.779	0.607
OAS1	0.800	0.158	0.665	0.796	0.633
CCL5	0.724	-0.331	0.634	0.734	0.539
IL1B	0.224	0.909	0.877	-	-
TGFB1	0.744	0.198	0.593	0.738	0.545

Supplementary Figure 1. Gene expression among individuals with different viral loads (VL). Lines represent the median with an interquartile range. p-values<0.05 were considered significant.

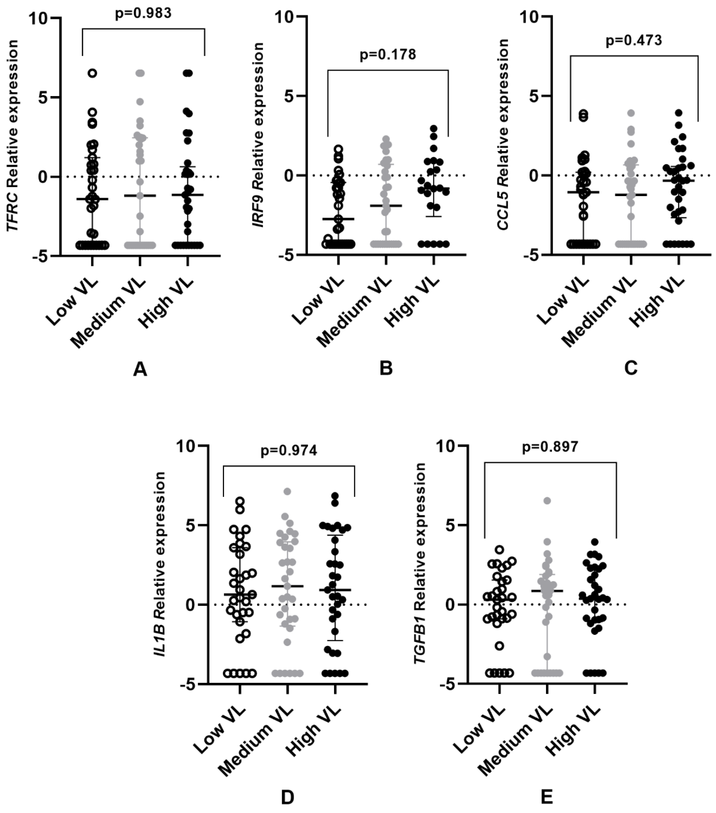

Supplementary Figure 2. Principal component analysis (PCA) plot. Genes were differently expressed between cases with COVID-19 and healthy controls and grouped into 2 components.

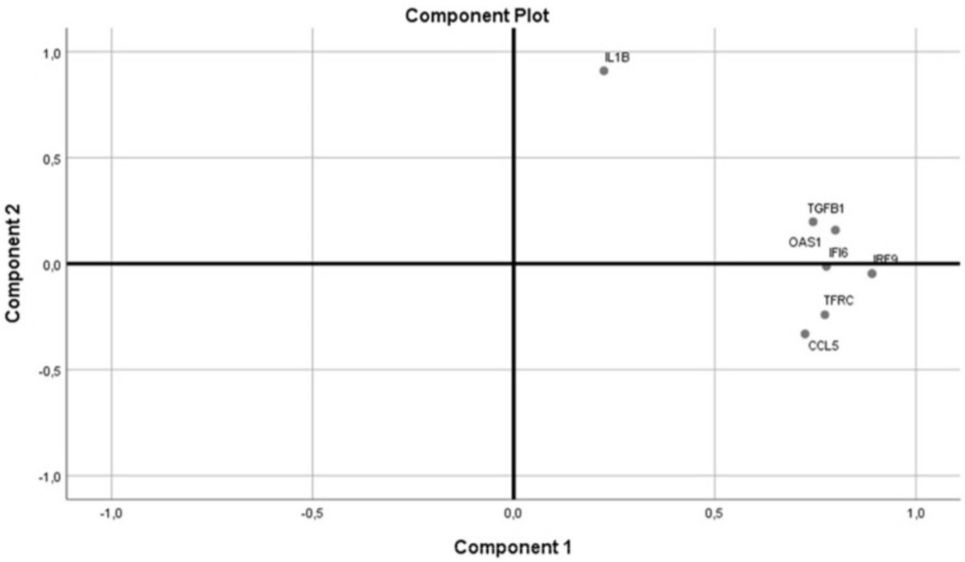

Supplement: Supplementary file 1 [file DataSheet_1.pdf]
